# Navigating the storm: How proficient organizational culture promotes clinician retention in the shift to evidence-based practice

**DOI:** 10.1371/journal.pone.0209745

**Published:** 2018-12-21

**Authors:** Nathaniel J. Williams, Rinad S. Beidas

**Affiliations:** 1 School of Social Work, Boise State University, Boise, Idaho, United States of America; 2 Department of Psychiatry, Perelman School of Medicine, University of Pennsylvania, Philadelphia, Pennsylvania, United States of America; 3 Department of Medical Ethics and Health Policy, Perelman School of Medicine, University of Pennsylvania, Philadelphia, Pennsylvania, United States of America; 4 Leonard Davis Institute of Health Economics, University of Pennsylvania, Philadelphia, Pennsylvania, United States of America; Institute of Mental Health, SINGAPORE

## Abstract

**Objective:**

Clinician turnover is a major concern as mental health systems and organizations invest substantial resources in the implementation of evidence-based practice (EBP). In this study, we identify malleable factors associated with reduced clinician turnover during a system-wide EBP implementation initiative. Specifically, we examine how proficient organizational culture (i.e., norms and behavioral expectations that clinicians prioritize improvement in client well-being and exhibit competence in up-to-date treatment practices), EBP implementation climate (i.e., perceptions that the organization’s policies, procedures, and practices support EBP use), and change in these organizational characteristics relate to clinician turnover during a system-wide EBP transformation.

**Method:**

Data were collected from 236 clinicians in 19 mental health clinics across 3 years of a system-wide EBP implementation initiative in the City of Philadelphia. Clinicians reported on proficient organizational culture and EBP implementation climate at baseline (T_1_) and two-year follow-up (T_2_). Administrators reported on clinician turnover at three-year follow-up (T_3_). Hypotheses were tested via multilevel mediation analyses incorporating mixed effects logistic regression models.

**Results:**

Controlling for organization size, clinician job satisfaction, attitudes towards EBP, job tenure, and age, higher levels of proficient organizational culture and improvement in proficient culture from baseline to two-year follow-up predicted reduced clinician turnover in the year following; these effects were mediated by EBP implementation climate and by improvement in EBP implementation climate, respectively.

**Conclusions:**

Organizations with more proficient cultures have more supportive EBP implementation climates that predict reduced clinician turnover during system-wide EBP implementation initiatives. Strategies that target these antecedents in mental health service organizations may contribute to reduced clinician turnover.

## Introduction

Employee turnover—that is, employees’ exit from the employment relationship—has long been recognized as a problem in organizations that deliver publicly-funded mental health services, with clinician turnover rates hovering around 30–60% annually in the United States [1, 2]. These high rates are associated with increased organizational costs, diminished organizational effectiveness [3, 4], breaches of clinician-client relationships [5, 6], reduced fidelity to evidence-based practices [7, 8], diminished expertise of the workforce, and poorer clinical outcomes [9]. Consequently, developing strategies to maintain a highly qualified and effective clinical workforce is a primary goal of efforts to improve the quality and outcomes of mental health care [10, 11].

In recent years, clinician turnover has taken on even greater significance with the advent of large-scale policy initiatives, regulatory mandates, and other efforts to improve the implementation of evidence-based practice in mental health service systems [12, 13]. Evidence-based practice (EBP) incorporates assessment, prevention, and intervention models that have demonstrated efficacy and effectiveness in well-conducted, rigorous research studies [14]. In an effort to improve the outcomes of clinical care, several States (e.g., Hawaii, New York, Oregon, Washington) and large service systems (e.g., Los Angeles County, Philadelphia) have recently enacted system-wide efforts designed to increase the delivery of EBP by mental health providers [15]. As part of these initiatives, systems and organizations invest substantial resources to train and support clinicians to deliver EBP [16]. When clinicians leave, these resources, and the clinician expertise associated with them, are lost even as additional costs are incurred to train and support new clinicians.

In order to reduce clinician turnover within the context of EBP implementation, knowledge is needed of the factors that support clinician retention during this significant system and organizational change. However, few studies have examined turnover within the context of EBP implementation [17]. Furthermore, the research conducted to date focuses on the *consequences* of clinician turnover during EBP implementation rather than *antecedents* [8], and few studies have examined turnover during naturalistic system-wide EBP implementation initiatives as opposed to randomized controlled trials [18, 19]. These are important gaps because understanding antecedents to turnover is critical to developing effective strategies and because the process of EBP implementation in community settings differs substantially from randomized trials [20].

In this study, we advance our understanding of mutable factors that mental health service organizations and systems can leverage to minimize clinician turnover in the midst of large-scale system-wide EBP implementation by focusing on organizational culture, defined as the integrated set of values, shared norms, and behavioral expectations that characterize and guide behavior within a work organization [1, 21]. Organizational culture is strongly linked to individual, group, and organizational outcomes [22] and experimental studies show that improvement in organizational culture contributes to improvement in the quality and outcomes of mental health services [23–25]. Theory suggests that specific types of organizational culture may also improve clinician retention, particularly as organizations respond to system-level EBP implementation initiatives [26–28]; however, no studies have empirically tested this relationship. Furthermore, the specific mechanisms through which culture influences clinician behavior are not well understood. Identification of mechanisms is important for developing effective strategies and for targeting strategies to specific settings [29].

The present study addresses these knowledge gaps by testing whether organizational culture is related to clinician turnover in mental health clinics during a system-wide EBP implementation initiative and by testing whether organizational culture’s relationship with clinician turnover is mediated by the development of specific organizational policies, procedures, and practices that provide clinicians with the support they need to successfully implement EBP [30]. Below, we review research that provides a basis for these hypotheses and we test them using data collected prospectively across three years of a system-wide EBP implementation initiative in the City of Philadelphia. If confirmed, our hypotheses have implications for reducing clinician turnover as systems and organizations transition to EBP.

### The multiple paths to employee turnover

The extensive literature on employee turnover has advanced significantly during the last 30 years, moving from narrow conceptualizations of turnover antecedents which characterized only a fraction of employee turnover experiences, to more complex models that explain the multiple paths employees take to terminating their employment [31, 32]. Among the most significant advances in this line of research are recognition of the roles played by shocks, which represent expected or unexpected events of either positive or negative valence that initiate the decision-making process involved in quitting a job [32], and image violations, which occur when an individual concludes that her or his values, goals, or strategies for goal attainment are incongruent with those of the employing organization [33, 34]. Recent studies indicate that shocks and image violations, which are especially likely to occur during times of significant organizational change, are a frequent cause of turnover, accounting for up to 60% of turnover experiences [35, 36]. Furthermore, shocks and image violations are particularly important antecedents to turnover in helping professions because of the importance placed by these professionals on personal values for motivating their work [37]. This research suggests that buffering against shocks and image violations may be key to reducing clinician turnover in general and during times of significant organizational and system change.

### Conceptualizing clinician turnover within the context of system-level EBP implementation initiatives

System-wide efforts to promote or mandate the implementation of EBP through policy initiatives, regulations, and fiscal measures represent a significant shock that may spur mental health clinicians to reconsider their employment—even if they welcome the requirement to modify their practice routines [36, 38]. Even more important, the way in which mental health service organizations respond to these efforts may constitute a secondary and even more powerful shock and may contribute to image violations, leading clinicians to re-evaluate their employment situation and potentially exit the organization [32].

Organizations have significant discretion in how they respond to large-scale EBP implementation initiatives and preliminary data suggests that misalignment between these responses and clinician values, goals, and strategies for goal attainment may lead to image violations and increased clinician turnover [17, 20]. For example, organizations that make only superficial changes to comply with system mandates while avoiding meaningful change (e.g., cosmetic modifications to documentation such as progress notes) or that pass the burden of complying with EBP implementation on to clinicians (e.g., requiring clinicians to complete EBP-related paperwork but not reimbursing for that time), may spur clinicians to re-evaluate the congruence between their personal values, goals, and strategies for goal attainment and those of their employing organization [17, 39]. Conversely, organizations that dedicate the resources necessary to determine how system-level EBP initiatives might be used to improve client well-being and provide clinicians with the support necessary to improve their competence and the message that recipients of services will benefit from EBP initiatives are likely to strengthen clinicians’ perceptions of the congruence between their personal values, goals, and strategies for goal attainment and those of the organization [38, 40]—potentially leading to decreased likelihood of turnover.

### Proficient culture as an antecedent to clinician retention

Studies of mental health services for youth indicate that organizational culture plays an important role in explaining how organizations approach service delivery in general and is likely to also influence how they respond to an EBP implementation initiative [28]. Organizational culture reflects the underlying values and assumptions that guide decision-making in an organization and as such shapes leadership’s responses to system-level policies and regulations [41]. Building on the research presented above, we propose that organizational culture will influence how clinicians’ experience a system-wide EBP implementation initiative and will lead to either increased or decreased likelihood of turnover during this significant system, organizational, and professional change.

We draw on an empirically-supported theoretical model of organizational culture proposed by Glisson and colleagues [42] to characterize the work environments of children’s mental health clinics. This model proposes that a specific type of organizational culture, referred to as *proficient* culture, contributes to improved service outcomes for youth and enhanced EBP implementation because it engenders norms and behavioral expectations that clinicians place the well-being of clients first, exhibit responsiveness to client needs, and maintain competence in up-to-date treatment models [26]. According to this model, clinicians do not respond directly to their work environment but rather to their shared *perceptions* of the organization’s norms and behavioral expectations [42]. Prior studies have shown that higher levels of proficient culture in child mental health clinics is linked to improved clinical outcomes for youth [43], more positive clinician work attitudes of job satisfaction and organizational commitment [26], better clinician attitudes towards EBP [44], and higher likelihood of clinicians’ adopting EBP [45] and achieving fidelity to EBP [46]. Experimental studies show that improvement in proficient culture contributes to increased clinician attendance at EBP workshops [24] and increased use of EBP with clients [27].

**Fig 1:**
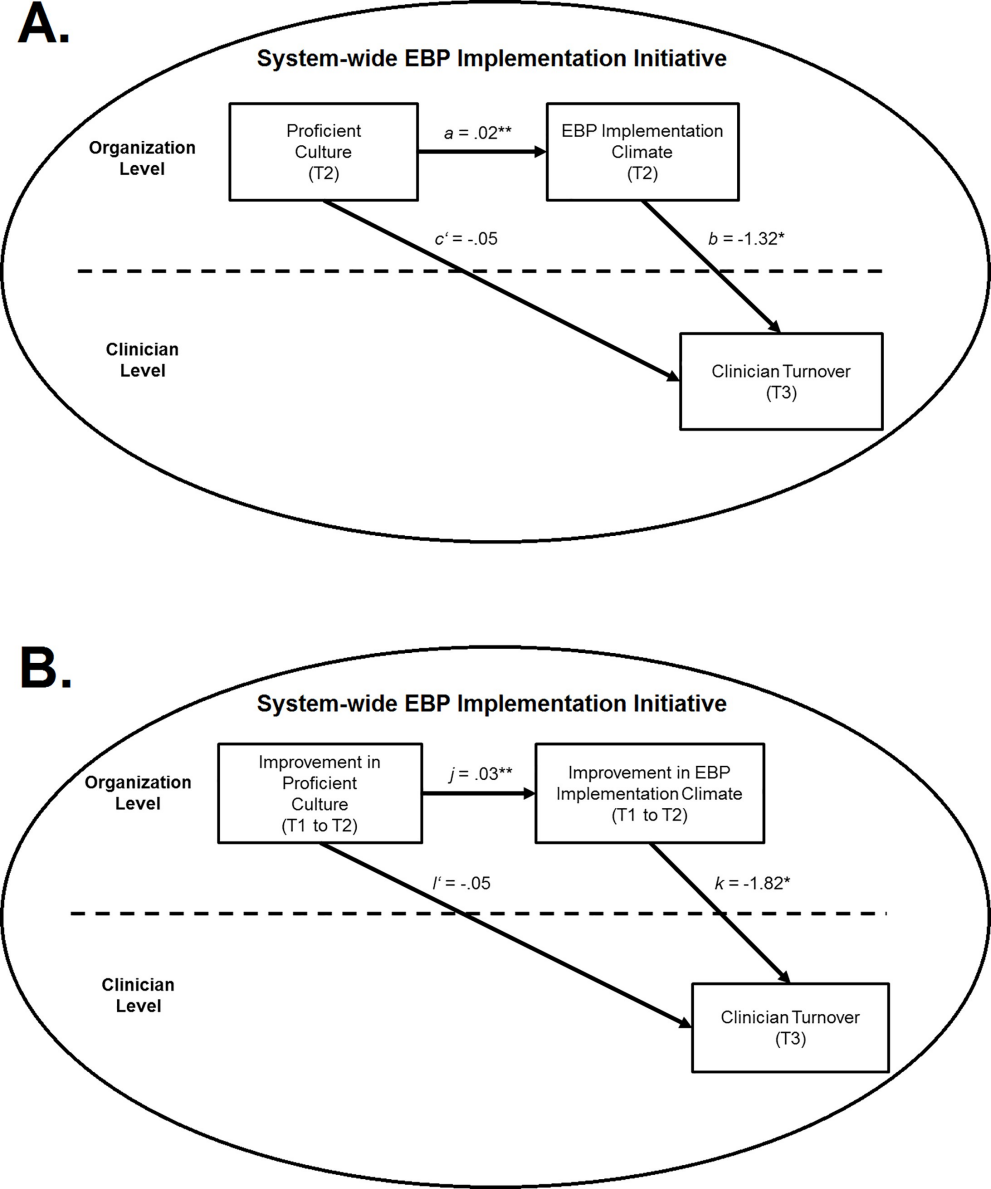
Hypothesized cross-level mediation models. *Note*: EBP = evidence-based practice; T1 = Time 1 (baseline); T2 = Time 2 (two-year follow-up); T3 = Time 3 (three-year follow-up). All models control for organization size, clinician job satisfaction, attitudes towards EBP, age, and job tenure. Indirect effects: a*b = -.03, *p* = .028, proportion mediated (p_m_) = .32; j*k = -.05, *p* = .004; p_m_ = .48. * *p* < .05. ** *p* < .01.

These studies suggest that highly proficient cultures are positively aligned with clinicians’ values, goals (e.g., focus on improvement in client well-being), and strategies for goal attainment (e.g., improved clinician competence) and therefore are likely to protect against clinician exits from the organization. Furthermore, this research suggests that highly proficient cultures will buffer against the potential image violations associated with system-wide EBP implementation initiatives by improving clinicians’ perceptions of EBP and supporting them in implementing EBP; consequently, proficient culture is likely to serve as a protective factor against turnover as organizations respond to system EBP implementation efforts. Fig 1A presents our theoretical model in which we hypothesize that higher levels of proficient organizational culture will serve as an antecedent to reduced clinician turnover within the context of a system-wide EBP implementation initiative.

### Implementation climate as a mediator

The presence of a proficient culture primes an organization to respond to a system-level EBP implementation initiative in a helpful way. However, the organization’s general support for clinician competence and improved client well-being must be translated into a *specific* and *targeted* set of policies, procedures, and practices that directly address the implementation of EBP [47]. The concept of *EBP implementation climate* describes clinicians’ shared perceptions that their organization enacts specific, strategically-focused policies, procedures, and practices that support, expect, and reward the use of EBP [30]. Examples of such policies and procedures include providing training, supervision, or release time to learn EBP, purchasing materials or providing specific space or equipment to implement EBP (e.g., workbooks, two-way mirror with adjacent rooms), and using expertise in EBP as a criterion for clinician promotion [38, 40]. Increased levels of EBP implementation climate are linked to improved clinician attitudes toward EBP [47] and EBP adoption [48] and potentially mediate the relationship between proficient culture and clinician turnover during system-wide EBP implementation efforts (see Fig 1A). We propose that higher levels of proficient organizational culture serve as an antecedent to higher levels of EBP implementation climate which in turn reduces the likelihood of clinician turnover within the context of a system-wide EBP implementation initiative. These hypotheses are presented in Fig 1A.

### Level and change in culture and climate as antecedents to retention

The research presented above suggests that clinicians who work in organizations with higher levels of proficient culture will be more likely to continue their employment during system-wide EBP implementation efforts and that these effects will be mediated in part or in whole by higher levels of EBP implementation climate (see Fig 1A). However, organizational culture can change over time [41], and to the extent that clinicians actively perceive and interpret these changes, it is likely that both the *level* and *change* in proficient culture and EBP implementation climate influence clinicians’ turnover during system-wide EBP implementation efforts. Building on this idea, we extend our theoretical model shown in Fig 1A to suggest that *improvement* in proficient culture from Time 1 to Time 2 will predict *improvement* in EBP implementation climate from Time 1 to Time 2 which will in turn predict reduced likelihood of individual clinician turnover at Time 3. Fig 1B shows these hypotheses. This type of effect, in which a positive *trajectory of change* in culture influences clinician behavior, has important implications for organizations that do not currently embody high levels of proficient culture.

### Study hypotheses

First, we hypothesized that clinicians working in organizations with more proficient cultures would be less likely to experience turnover during the following year of a system-wide EBP implementation initiative, controlling for clinician job satisfaction and other general factors that predict turnover. Second, we hypothesized that higher levels of EBP implementation climate would mediate the relationship between proficient culture and reduced clinician turnover as is shown in Fig 1A. Third, we hypothesized that, controlling for proficient culture at baseline and other covariates, clinicians in organizations where proficient culture demonstrated greater improvement over 2-years of an EBP implementation initiative would be less likely to experience turnover in the following year compared to clinicians in organizations where proficient culture was less improved. Fourth, we hypothesized that improvement in EBP implementation climate would mediate the relationship between improvement in proficient culture and reduced clinician turnover as is shown in Fig 1B.

## Method

### Setting

To test our hypotheses, we conducted a prospective study of clinician turnover within the context of a large-scale EBP system transformation within the City of Philadelphia. Beginning in 2007, the Philadelphia Department of Behavioral Health and Intellectual disAbility Services (DBHIDS) launched a number of system-level activities designed to promote the use of EBP by organizations participating in its Medicaid-funded mental health provider network. A central goal was to bring the best available science to community treatment. Given the state of the research, the primary models deployed to date have focused on interventions stemming from the cognitive behavioral orientation. Cognitive-behavioral therapy (CBT) has extensive evidence of effectiveness for the treatment of the most common psychiatric disorders of childhood and adolescence [14]. Early system-level activities included delivering training to clinicians in a variety of CBT approaches through a series of independent training and consultation initiatives [16].

In 2013, DBHIDS intensified and formalized these efforts through the creation of a centralized infrastructure called the Evidence-Based Practice and Innovation Center (EPIC). The launch of EPIC marked a major systemic shift from isolated efforts to encourage EBP use, to an integrated and concerted set of formal activities designed to increase EBP implementation within the provider network [49]. EPIC falls under the auspices of Community Behavioral Health (CBH), a not-for-profit 501c (3) corporation that contracts with DBHIDS to provide mental health services for Philadelphia County Medicaid recipients. Its activities include: (a) promoting EBP to leaders of mental health organizations, clinicians, and consumers within their network; (b) overseeing EBP clinical training and consultation to clinicians within the network; (c) building organizational capacity to deliver EBP through operational support and technical assistance; and, (d) leveraging financing models (e.g., enhanced reimbursement rates for EBP use) to support EBP implementation within the network [49]. All organizations within the CBH network had access and equivalent opportunities to engage with EPIC. Additionally, DBHIDS provided foundational training in EBP to all employees of DBHIDS and CBH in 2014. This coordinated, system-level EBP implementation effort, enacted by the primary funder (CBH) and regulator (DBHIDS) of public mental health services in the Philadelphia system, provided a natural laboratory within which to study how variation in organizations’ cultures and climates prospectively related to clinician turnover during major system-level shifts to EBP.

### Participants

The study sample included 236 mental health clinicians working in 19 outpatient mental health organizations (*M* clinicians per organization = 12.42, min = 4, max = 40). Of the approximately 100 organizations that deliver youth mental health services within the Philadelphia system, 29 of the largest child-serving mental health organizations were invited to participate. Of these, 18 (62%) agreed to participate and one additional organization requested to participate, resulting in a sample of 19 organizations at baseline. Several organizations had multiple sites with distinct locations and leadership structures representing a total of 23 sites which we treated as distinct organizations at baseline (we refer to sites as organizations in the remainder of the paper). Nineteen of these organizations (83%) were retained at three-year follow-up and had three or more clinicians for whom turnover could be observed during the third year of the study (*k* = 2 organizations were no longer in operation; *k* = 1 organization declined to participate; *k* = 1 organization had < 3 participating clinicians at Time 2). We based our inclusion criteria of three or more clinicians per organization on methodological research showing that the inclusion of all organizations with three or more respondents in the analysis improves the accuracy of statistical inferences and optimizes statistical power [50, 51].

Within participating organizations, all clinicians who delivered direct mental health services to youth were invited to participate (*M* within-organization response rate = 60%, min = 23%, max = 92%). On average, participating clinicians were 38.67 years old (*SD* = 11.89), had several years of experience delivering services (*M* = 8.38 years of experience, *SD* = 7.56), and had considerable tenure in their current organization (*M* = 3.03 years; *SD* = 4.18). Clinicians were primarily female (*n* = 179, 76%), had graduate degrees in social work, counseling, or allied health fields (*n* = 220, 93%), and came from heterogeneous ethnic and racial backgrounds (43.6% White, 30.5% African American, 12.3% Hispanic/Latino, 4.7% Multiracial, 4.7% Asian, and 2.1% “Other,” with 6 individuals not reporting).

### Procedures

Data collection occurred in three waves corresponding to baseline (2013), two-year follow-up (2015), and three-year follow-up (2016). Baseline data were collected during the first 6 months of EPIC’s system-level EBP activities [49]. By the two-year follow-up, EPIC had sponsored foundational training in EBP for all employees of DBHIDS and CBH, overseen multiple rounds of provider training and technical assistance in EBP, implemented an enhanced reimbursement rate for the delivery of one specific EBP, created a communication infrastructure (i.e., website and email list), informally provided support to leadership of agencies implementing EBP, and convened two annual EBP showcase conferences, among other activities (see [49]). By the three-year follow-up, EPIC’s system-wide activities continued at full capacity, and the one-year lag provided an opportunity to assess the likelihood of observed clinician turnover.

At Times 1 and 2, clinicians completed questionnaires reporting on organizational culture and climate, job satisfaction, EBP attitudes, and demographic information. Questionnaires were completed during a 2-hour meeting at clinicians’ places of employment during regular work hours without organizational leaders present. In the meetings, researchers obtained written informed consent, provided lunch, and administered the questionnaires. Participants were provided with $50. At Time 3, researchers contacted the administrators at each organization, provided them with a list of clinicians who were present one year earlier (i.e., at Time 2), and asked them to identify all clinicians who had left the organization in the 12 months prior. This process resulted in collection of turnover data for all *n* = 165 participating clinicians who were present at Time 2. All data collection procedures were approved by the institutional review boards of the University of Pennsylvania and the City of Philadelphia.

### Measures

#### Proficient organizational culture

We measured proficient organizational culture using the 15-item *proficiency* scale of the Organizational Social Context (OSC) measure [26]. Scores on the proficiency scale have demonstrated excellent reliability, structural validity, criterion-related validity, and predictive validity [26, 43–46]. Items on the proficiency scale refer to shared organizational norms and behavioral expectations that clinicians place the well-being of clients first (i.e., responsiveness), and that clinicians are competent and have up-to-date knowledge of effective treatment practices (i.e., competence). Confirmatory factor analysis indicates the two sub-dimensions load onto a single latent factor represented by the proficiency total score [26]. Item responses are made on a five-point scale ranging from 1 (*Never*) to 5 (*Always*). Coefficient alpha for this scale was α = .92 at baseline and α = .93 at two-year follow-up.

#### EBP implementation climate

The level of EBP implementation climate was measured using the 18-item Implementation Climate Scale (ICS) [30]. The ICS is designed to assess clinicians’ shared perceptions that the organization’s policies, procedures, and practices support and reward EBP implementation. The total score is derived from items addressing six sub-dimensions relating to the organization’s focus on EBP, educational support for EBP, rewards and recognition for EBP, and selection of staff for EBP expertise and openness. Consistent with prior research and with our interest in the overall EBP implementation climate, we calculated the mean score across all six dimensions. Evidence of structural, convergent, and discriminant validity for the ICS total score is strong [30]. Responses on the ICS are made on a 0 (*Not at All*) to 4 (*A Very Great Extent*) scale. Coefficient alpha for this scale was α = .94 at baseline and α = .95 at two-year follow-up.

#### Clinician turnover

For all mental health clinicians present at Time 2 (*n* = 165), we determined whether they had left the organization or remained employed with their organization 12 months later by contacting administrators at Time 3. Employment status changes involving promotions to supervisory positions or transitions to other positions within the organization were not counted as turnover events because the EBP expertise and training represented by the clinician remained within the organization.

#### Control variables

In order to eliminate potential confounds and to improve statistical power, we selected organization- and clinician-level covariates to include as control variables in our analyses on the basis of theory and prior research. The following variables were included as controls in our analyses:

*Clinician job satisfaction* is a well-established predictor of turnover that reflects clinicians’ overall positive or negative appraisal of their job and job experiences [31]. We included job satisfaction in our models to provide discriminant validity evidence for the effects of proficient organizational culture and EBP implementation climate independent of clinicians’ general satisfaction with their job experiences. Job satisfaction was measured using five well-established items developed and validated specifically in youth mental health service settings and included in the Organizational Social Context (OSC) measure [26]. Coefficient alpha for this scale was α = .72 in the present study.

*Clinician attitudes towards evidence-based practice* is defined as a clinician’s overall appraisal of whether the use of EBP is positive or negative and was measured using the well-established, 15-item Evidence-Based Practice Attitudes Scale (EBPAS) [52]. The EBPAS includes four subscales that assess clinicians’ willingness to adopt an EBP based upon its appeal or requirements from external sources, general openness to innovation, and perceptions of divergence between their current practice and EBP. We included this covariate to provide discriminant validity evidence to support the importance of proficient culture and implementation climate apart from clinicians’ individual attitudes towards EBP. Coefficient alpha for this scale was α = .75 in the present study.

Two clinician demographic characteristics were included as covariates in our models based on an extensive body of research showing that these variables are consistently related to employee turnover [34]: *job tenure*, measured as clinicians’ self-reported years present in their current organization, and *age*, measured as clinicians’ self-reported age in years.

At the organizational level we included *organization size*, measured as the total number of therapists employed by the organization as reported by clinic leaders, as a covariate because prior research has shown that organization size relates to employee turnover and because larger mental health clinics likely have greater resources and capacity to support employees during the transition to EBP [53].

### Data aggregation

Consistent with best practices in organizational and multilevel research [26, 54], we generated organization-level values for proficient organizational culture and EBP implementation climate by aggregating (i.e., averaging) clinicians’ individual responses to the proficiency and ICS scales, respectively. Aggregation of these scores reflects the fact that both variables are conceptualized as social characteristics of organizations and not as characteristics of individuals. The items in these scales refer to the organization (e.g., “Clinicians in my organization are expected to …”) and are designed to be aggregated once evidence of within-organization agreement has been demonstrated [26, 54]. For both variables, aggregation was based on evidence of within organization agreement provided by calculation of *r*_wg(j)_ values with a uniform null distribution [54, 55]. For proficient culture the mean *r*_wg(j)_ at baseline was .97 (range .94 to .99) and at wave 2 it was .96 (range .86 to .99). For EBP implementation climate the mean *r*_wg(j)_ at baseline was .91 (range .79 to .99) and at wave 2 it was *r*_wg(j)_ = .92 (range .80 to .99). All values were above the standard cutoff value of .70 [56].

#### Data analytic approach

Two critical features of this study are the nested data structure (i.e., clinicians within organizations) and the cross-level hypotheses. To address these features we relied on two-level mixed-effects logistic regression models with random organization intercepts implemented via the TWOLEVEL procedure in Mplus software, version 7 [57, 58]. These models test the cross-level effects of proficient organizational culture and change in proficient culture on clinicians’ logged odds of turnover controlling for organizational and clinician covariates. Missing data on clinician-level predictor variables was less than 2% and were imputed using the serial mean. All covariates were grandmean centered in order to adjust the models for organization-level differences in clinician composition [58]. Preliminary analyses supported our multilevel approach by providing evidence of significant between-organization variance in clinician turnover (η^2^ = .29, *p* < .001).

To test our cross-level mediation hypotheses, we relied on multilevel path analysis implemented via the TWOLEVEL procedure in Mplus, version 7 [57]. Multilevel path analysis is a special case of multilevel structural equation modeling that permits the specification and testing of cross-level indirect effects with covariates at the clinician (level 1) and organization (level 2) levels [59]. In Mplus, multilevel path analysis is implemented using a robust maximum likelihood estimation (MLR) algorithm that accommodates missing data, unbalanced cluster sizes, and random organization intercepts [59]. Because of our dichotomous outcome at level 1, the models also incorporated a logit link function in the second step of the analysis (i.e., two-level mixed effects logistic regression models).

The indirect effects tested in this study incorporate an organization-level independent variable, an organization-level mediator, and a clinician-level outcome, also referred to as 2-2-1 mediation, representing the level of each construct in the analysis [60, 61]. As is shown in Fig 1, the product of the *a* and *b* paths (i.e., a*b) or the *j* and *k* paths (i.e., j*k), respectively, estimates the cross-level indirect effect of proficient culture (or change in proficient culture) on clinician turnover via EBP implementation climate (or change in EBP implementation climate).

## Results

Forty percent of clinicians (*n* = 66) left their organization during the 12-month observation period in the third year of the study. Rates of clinician turnover varied significantly across organizations, χ^2^(18) = 47.35, *p* < .001, ranging from 0% for three organizations to 100% for one organization. Approximately half of the organizations (*k* = 9, 47%) had turnover rates ≥ 50% and the average turnover rate within these organizations was 67%. For organizations with turnover rates < 50% (*k* = 10), the average turnover rate was 18%. The significant between-organization variance in turnover rates suggests that organizational characteristics played a role in explaining clinician turnover during the study period.

Table 1 presents descriptive statistics and correlations for the study variables. Consistent with our study hypotheses, higher levels of proficient culture and EBP implementation climate at Time 2 were associated with reduced clinician turnover at Time 3 (*r* = -.30 and *r* = -.35, respectively, *p*s < .05). Furthermore, there was evidence that proficient culture and EBP implementation climate were related but distinct constructs, *r* = .47, *p* = .041. Also consistent with prior research, higher levels of proficient culture were related to higher levels of job satisfaction (*r* = .43, *p* < .001) and higher job satisfaction was related to reduced clinician turnover (*r* = -.21, *p* = .006). The correlation matrix also indicated that older clinicians (*r* = -.22, *p* = .004) and clinicians who had better attitudes towards EBP (*r* = -.18, *p* = .025) were less likely to exit their organizations.

**Table 1:** Descriptive statistics and correlations for study variables.

Variable	Mean	SD	Min.	Max.	1	2	3	4	5	6	7	8	9
Organization Level													
1. Proficient culture (T1)	49.08	13.28	12.52	66.75									
2. Proficient culture (T2)	55.12	9.61	26.01	70.07	.34								
3. EBP implementation climate (T1)	1.98	.46	1.11	3.23	.57*	.04							
4. EBP implementation climate (T2)	1.98	.48	1.22	2.98	.25	.47*	.35						
5. Organization size (T2) (# therapists)	13.00	10.96	4	55	-.15	-.20	-.36	-.25					
Clinician Level													
6. Age in years (T2)	39.21	12.10	23	76	-.01	.01	.19*	.15	-.02				
7. Job tenure in years (T2)	3.15	4.11	0	30	-.09	-.02	.02	.07	-.11	.42**			
8. Job satisfaction (T2)	18.52	3.59	6	25	.34**	.43**	.32**	.40**	-.25**	.03	.01		
9. EBP attitudes (T2)	2.94	.47	1.44	3.94	.02	.02	-.02	.02	-.06	.01	-.06	.03	
10. Turnover (T3) (yes = 1)	.40	.49	0	1	-.02	-.30**	-.14	-.35**	.15	-.22**	-.10	-.21**	-.18*

*Note*: *k* = 19 organizations, *n* = 165 clinicians; EBP = evidence-based practice; T1 = Time 1 (baseline), T2 = Time 2 (two-year follow-up), T3 = Time 3 (three-year follow-up).

* *p* < .05

** *p* < .01

**Fig 2:**
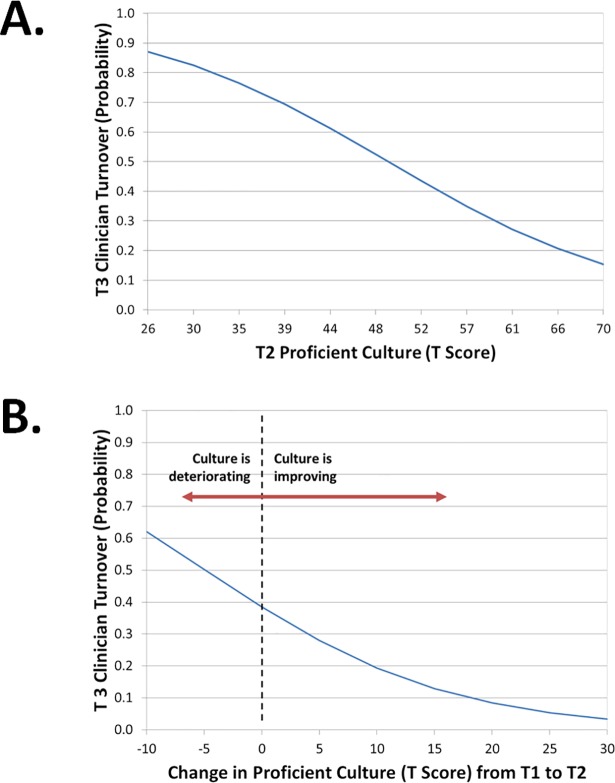
Relationship between proficient organizational culture, change in proficient culture, and probability of clinician turnover during the following 12 months. *Note*: T Score *μ* = 50, *σ* = 10. T1 = Time 1 (baseline); T2 = Time 2 (two-year follow-up); T3 = Time 3 (three-year follow-up). All models control for organization size, clinician job satisfaction, attitudes towards EBP, age, and job tenure.

Hypothesis 1 stated that clinicians working in organizations with more proficient cultures would be less likely to exit their organization in the following year of a system-wide EBP implementation initiative, controlling for clinician job satisfaction and other factors. Results of the mixed effects logistic regression analysis supported this hypothesis (see Table 2). Controlling for organization size, clinicians’ job satisfaction, EBP attitudes, job tenure, and age, clinicians in more proficient organizational cultures were less likely to exit the organization during the following 12 months, *OR* = .921, *p* = .044 (see Table 2). As is shown in Fig 2A, the probability of clinician turnover in organizations that were one standard deviation *below* the mean on proficient culture T-score (turnover probability = .58) was 2.6 times as great as that of clinicians in organizations one standard deviation *above* the mean on proficient culture T-score (turnover probability = .22). Further, the turnover probability of .87 for clinicians in the *least* proficient culture in this sample was 5.8 times as great as the turnover probability for clinicians in the *most* proficient culture in this sample (turnover probability = .15). These results confirm that proficient culture is a robust predictor of the likelihood of clinician turnover during system-wide EBP initiatives even after controlling for job satisfaction, EBP attitudes, and other covariates.

**Table 2:** Two-level mixed effects logistic regression analyses predicting clinician turnover.

	Clinician Turnover (T3)					
		95% CI			95% CI	
Predictor	*OR*	LL	UL	*OR*	LL	UL
Clinician Level						
Job tenure (T2)	.991	.892	1.102	.995	.899	1.102
Age (T2)	.964**	.941	.987	.963**	.940	.986
Attitudes toward EBP (T2)	.411	.159	1.059	.409	.158	1.057
Job satisfaction (T2)	.917	.825	1.019	.909	.820	1.009
Organizational Level						
Org. size (# therapists) (T2)	.994	.969	1.020	.997	.972	1.023
Proficient culture (T2)	.921*	.850	.998	.908*	.832	.992
Proficient culture (T1)				1.036	.980	1.094
*Pseudo-R*^*2*^	.47			.59		

*Note*: *k* = 19 organizations, *n* = 165 clinicians; CI = confidence interval; EBP = evidence-based practice; T1 = Time 1 (baseline), T2 = Time 2 (two-year follow-up), T3 = Time 3 (three-year follow-up); OR = odds ratio; LL = lower limit; UL = upper limit. *Pseudo-R*^*2*^ calculated as (*τ*_null–_ *τ*_model_) / *τ*_null_

* *p* < .05

** *p* < .01

Hypothesis 2 stated that higher levels of EBP implementation climate would mediate the relationship between proficient organizational culture and reduced clinician turnover. As is shown in Fig 1A, results of the multilevel mediation analysis supported this hypothesis. In the first step of the analysis (i.e., path *a*), higher levels of proficient organizational culture predicted increased EBP implementation climate, *B* = .02, *SE* = .01, *p* = .004, controlling for covariates, accounting for 25% of the variance in implementation climate (see Table 1). In the second step of the analysis (i.e., path *b*), higher levels of EBP implementation climate predicted decreased clinician turnover during the following year, *OR* = .27, *p* = .043, controlling for covariates and proficient culture (see Fig 1A). The indirect effect in this analysis was statistically significant (a*b = -.03, *p* = .028) confirming that the association between proficient organizational culture and reduced clinician turnover was mediated by higher levels of EBP implementation climate. The proportion mediated statistic [62] calculated as (a*b)/*c* indicated that EBP implementation climate explained 32% of the relationship between proficient culture and reduced clinician turnover.

Hypothesis 3 stated that, controlling for proficient culture at baseline, clinicians in organizations where proficient culture had exhibited greater improvement over 2-years of a system-level EBP implementation initiative would be less likely to turnover than clinicians in organizations where proficient culture had shown less improvement. Results of the mixed effects regression analysis supported this hypothesis. Controlling for organization size, clinician job satisfaction, EBP attitudes, job tenure, age, and proficient culture at baseline, improvement in proficient culture (as represented by the Time 2 proficient culture T score) predicted decreased clinician turnover, OR = .91, *p* = .032 (see Table 2). As is shown in Fig 2B, clinicians in organizations where proficient culture had improved during the two-year period were less likely to turnover in the subsequent 12-months than clinicians in organizations where proficient culture was unchanged or had deteriorated. Specifically, in organizations that improved by six points on proficient culture T score, which represented the average level of change in this sample (and approximately one-half of a population standard deviation), the probability of clinician turnover decreased by 32% relative to organizations where the level of proficient culture remained unchanged. These results confirm that both the *level* of proficient culture and the *change* in proficient culture during the preceding two years predicted the likelihood of clinician turnover during this system-wide EBP transformation, after controlling for organizational and clinician covariates.

Hypothesis 4 stated that improvement in EBP implementation climate would mediate the relationship between improvement in proficient organizational culture and reduced clinician turnover. Results of the multilevel mediation path analysis supported this hypothesis. Controlling for baseline proficient culture and baseline EBP implementation climate, as well as the other covariates, improvement in proficient organizational culture from baseline to two-year follow-up (as represented by the Time 2 proficient culture T score) predicted improvement in EBP implementation climate from baseline to two-year follow-up (as represented by the Time 2 score), *B* = .03, *SE* = .01, *p* = .002, accounting for 35% of the variance (see Fig 1B). Furthermore, improvement in EBP implementation climate predicted decreased clinician turnover during the following 12 months, OR = .16, *p* = .011, controlling for baseline proficient culture, baseline EBP implementation climate, improvement in proficient culture and the other covariates (see Fig 1B). The indirect effect in this mediation analysis was statistically significant (j*k = -.05, *SE* = .016, *p* = .004) confirming that improvement in proficient culture reduced clinician turnover indirectly through improvement in EBP implementation climate after controlling for covariates. Improvement in EBP implementation climate accounted for 48% of the effect of improvement in proficient culture on reduced clinician turnover.

## Discussion

Clinician turnover represents a significant threat to the implementation of EBP and to mental health service effectiveness generally [38]. In this study, we examined how proficient organizational culture related to clinician turnover during a system-wide EBP implementation initiative. Further, we tested EBP implementation climate as a mechanism linking proficient culture to reduced clinician turnover. We conceptualized EBP implementation as a shock that introduces the potential for clinician image violations and turnover [31] as clinicians in less proficient cultures witness maladaptive organizational responses to system-level EBP initiatives. Results supported our hypotheses; the probability of clinician turnover was 5.8 times as great in the least proficient culture compared to the most proficient culture and 32% of this effect was mediated by higher levels of EBP implementation climate. Furthermore, and perhaps most importantly, the amount of improvement in proficient culture during two years of the initiative also predicted reduced clinician turnover and 48% of this effect was explained by improvement in EBP implementation climate, suggesting that improvement in these organizational characteristics also supports clinician retention. These results have important implications as systems and organizations seek to reduce clinician turnover during the transition to EBP.

Results of this study suggest organizations can reduce clinician turnover during system-wide EBP implementation efforts by developing proficient cultures. Proficient organizational culture is an attractive target for strategies to reduce turnover because it is malleable, feasible, influences many clinicians simultaneously (i.e., has broad each), and is positively associated with other implementation outcomes [25, 27]. Perhaps most importantly, the fact that there were a group of organizations in this sample that demonstrated highly proficient cultures (despite being exposed to the same external policy, regulatory, and fiscal constraints as less proficient organizations in the same sample) suggests it is possible to build proficient cultures in organizations [17, 27, 46]. Evidence of the malleability and feasibility of changing proficient culture also stems from research demonstrating that proficient culture can be improved and that these improvements explain the effect of organizational implementation strategies on clinicians’ EBP exploration, adoption, and use [25, 27]. These studies suggest that proficient culture can be improved in 18 months and that these changes support EBP implementation. In addition to these outcomes, experiments are needed to test whether intervening on proficient culture contributes to reduced clinician turnover. Future research should also examine the specific types of leadership that serve as an antecedent to the formation of a proficient culture and whether there are indirect effects of leadership on turnover through proficient culture [63].

So how do organizations and systems create proficient cultures? Researchers are beginning to experiment with different approaches. One evidence-based intervention for developing proficient cultures is the Availability, Responsiveness, and Continuity organizational strategy [24, 64]; however, this intervention is time and resource intensive (e.g., 3 years) and may benefit from additional development to increase its feasibility. Other interventions that focus on leaders of provider organizations may also be beneficial. In the interim, findings from this study indicate that measurement of proficient culture prior to EBP implementation may assist in identifying organizations most at-risk for experiencing difficulties with clinician retention during this system change.

In addition to illuminating the protective effect of proficient culture, this study sheds light on one mechanism that may link proficient culture to reduced clinician turnover amidst system-wide EBP transformations—EBP implementation climate. Organizations can develop strong EBP implementation climates through a range of policies and procedures including education to support EBP (e.g., training, supervision, peer consultation), recognition for clinicians who develop expertise in EBP, and rewards (e.g., financial incentives) for use of EBP [30, 47]. Studies are underway to test implementation strategies that generate supportive EBP implementation climates in substance abuse treatment centers [65] and this research should be expanded to mental health settings as well.

Even if organizations did not initially exhibit highly proficient cultures or EBP implementation climates, *improvement* in proficient culture and EBP implementation climate exhibited a protective relationship with clinician turnover, suggesting that positive change in these areas can support clinician retention. This finding highlights the importance of assessing these constructs prior to engaging in implementation efforts and continuing to assess them over time, given that actively attending to positive or negative changes in organizational culture and climate during the process of implementation has the potential to serve a protective effect with regard to turnover.

This study has a number of strengths and limitations. First, because our study was conducted within the context of a naturalistic, system-wide EBP implementation initiative we cannot make causal inferences. The prospective study design provides increased confidence in the results by establishing the temporal precedence of proficient culture and EBP implementation climate relative to clinician turnover; however, our results cannot conclusively establish the direction of this relationship. Most theories have described how organizational culture might cause variation in individual turnover; however, theory development is needed to explore the potentially bidirectional relationship between individual clinician turnover and organizational culture. Most importantly, experimental studies are needed to test whether change in proficient culture and EBP implementation climate serve as mechanisms for increasing clinician retention.

Second, the independent measurement of organizational culture and climate by clinicians versus the observation of clinician turnover by administrators increases confidence in the study findings. Self-report measures are sometimes viewed as a weakness of research; however, in this case perceptual measures of organizational culture and climate are required by theory because clinicians respond to their perceptions of the social environment not to the actual social environment *per se*.

Third, studies of mediation ideally show that change in the independent variable preceded change in the mediator which ultimately contributed to change in the outcome [29]. Difficulties in obtaining large samples of mental health provider organizations that are all subject to the same policy environment makes it difficult to test such granular level hypotheses in mental health services [49]; however, this study incorporates features that increase confidence in mediation including the temporal lag between the antecedents and the outcome as well as the demonstration that change in the independent variable predicted change in the mediator which ultimately predicted variation in the outcome.

Fourth, we did not have information on voluntary vs. involuntary turnover nor on the specific reasons clinicians exited the organization (i.e., related to EBP implementation or some other reason). Meta-analytic research indicates that while voluntary turnover is consistently related to worse organizational performance, involuntary turnover has neither a consistently positive nor negative relationship with performance [4]. In mental health services, the vast majority (e.g., 79–88%) of clinician turnover is voluntary [8, 17] and studies have shown that both voluntary and involuntary clinician turnover are negatively related to EBP fidelity [7, 8]; consequently, identifying variables that reduce both types of turnover is important. Our study takes a first step in that direction and future research could examine whether proficient culture is more strongly related to voluntary vs. in voluntary turnover. Further, we did not assess clinicians’ specific reasons for leaving the organization and consequently we cannot be sure whether clinicians’ left because of the implementation initiative or for some other reason. Future studies should assess clinicians’ reasons for leaving and evaluate how these relate to turnover.

Fifth, it is possible that other variables not included in our analyses are related to clinician turnover. The analytic models presented here incorporate what we believe are the most important potential confounds, including clinicians’ job satisfaction, attitudes toward EBPs, job tenure, age, and organization size; however, additional studies are needed to replicate these findings and to rule out alternative potential confounds.

Results from this study identify two novel, malleable, and feasible targets that systems and organizations can engage in efforts to improve clinician retention during system-wide EBP implementation initiatives. By generating proficient organizational cultures and supportive EBP implementation climates, systems and organizations can optimize their return on investment in EBP implementation initiatives and provide the best opportunity to improve mental health service outcomes for youth.
